# Bis{μ-4,4′-dimeth­oxy-2,2′-[propane-1,2-diylbis(nitrilo­methyl­idyne)]diphenolato}bis­({4,4′-dimeth­oxy-2,2′-[propane-1,2-diylbis(nitrilo­methyl­idyne)]diphenol}manganese(III)) bis­(hexa­fluorido­phosphate)

**DOI:** 10.1107/S1600536809028591

**Published:** 2009-07-29

**Authors:** Mohammad Hossein Habibi, Elham Askari, Reza Mokhtari, Morteza Montazerozohori, Takayoshi Suzuki

**Affiliations:** a Catalysis Division, Department of Chemistry, University of Isfahan, Isfahan 81746-73441, Iran; b Department of Chemistry, Yasouj University, Yasouj, 75914-353, Iran; c Department of Chemistry, Faculty of Science, Okayama University, Tsushima-naka 3-1-1, Okayama 700-8530, Japan

## Abstract

In the title complex, [Mn_2_(C_19_H_20_N_2_O_4_)_2_(C_19_H_22_N_2_O_4_)_2_](PF_6_)_2_, the Mn^III^ ion is coordinated by two O [Mn—O = 1.855 (2) and 1.887 (2) Å] and two N [Mn—N = 1.982 (3) and 1.977 (3) Å] atoms from the tetra­dentate Schiff base ligand and a coordinated axial ligand [Mn—O = 2.129 (2) Å]. The centrosymmetric dimer contains two Jahn–Teller-distorted Mn^III^ ions, each in a nearly octa­hedral geometry, connected through two phenolate bridges from two ligands. There are two stereogenic centers. The methyl group and the H atom attached to the middle propane C atom are disordered over two positions with occupancy factors in the ratio 0.58:0.42. The crystal is therefore a mixture of two diasteroisomers, *viz. RS/SR* and *RR/SS*. In the axial ligand, the two benzene rings form a dihedral angle of 56.97 (5)° and the dihedral angle between the two MnNC_3_O chelate rings is 2.98 (12)°

## Related literature

For general background to Schiff bases, see: Vites & Lynam (1998 ▶); Pecoraro & Butler (1986 ▶); Antonyuk *et al.* (2000 ▶); Barynin *et al.* (2001 ▶); Meier *et al.* (1996 ▶); Stemmler *et al.* (1997 ▶); Glatzel *et al.* (2004 ▶); Dixit & Srinivasan (1988 ▶); Lu *et al.* (2006 ▶); Stallings *et al.* (1985 ▶). For related structures, see: Habibi *et al.* (2007*a* ▶,*b* ▶,*c* ▶); Eltayeb *et al.* (2008*a* ▶,*b* ▶); Mitra *et al.* (2006 ▶). For bond-length data, see: Allen *et al.* (1987 ▶).
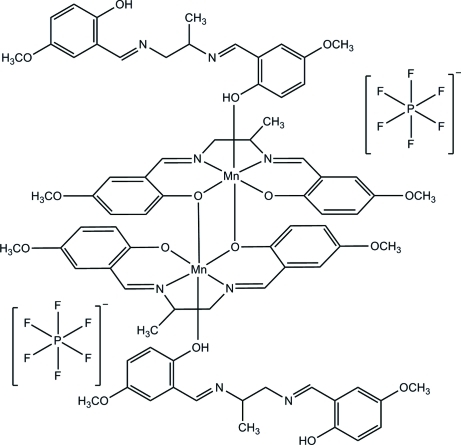


## Experimental

### 

#### Crystal data

[Mn_2_(C_19_H_20_N_2_O_4_)_2_(C_19_H_22_N_2_O_4_)_2_](PF_6_)_2_
*M* *_r_* = 1761.30
Monoclinic, 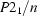

*a* = 13.2754 (5) Å
*b* = 22.658 (1) Å
*c* = 14.0774 (5) Å
β = 111.476 (1)°
*V* = 3940.4 (3) Å^3^
*Z* = 2
Mo *K*α radiation
μ = 0.46 mm^−1^
*T* = 200 K
0.26 × 0.12 × 0.08 mm

#### Data collection

Rigaku R-AXIS RAPID diffractometer
Absorption correction: numerical (*ABSCOR*; Higashi, 1995 ▶) *T*_min_ = 0.890, *T*_max_ = 0.964
60946 measured reflections
8971 independent reflections
5923 reflections with *I* > 2σ(*I*)
*R*_int_ = 0.054

#### Refinement

*R*[*F* ^2^ > 2σ(*F* ^2^)] = 0.070
*wR*(*F* ^2^) = 0.219
*S* = 1.08
8971 reflections
539 parameters
2 restraints
H-atom parameters constrained
Δρ_max_ = 0.73 e Å^−3^
Δρ_min_ = −0.62 e Å^−3^

### 

Data collection: *PROCESS-AUTO* (Rigaku, 1998 ▶); cell refinement: *PROCESS-AUTO*; data reduction: *CrystalStructure* (Rigaku/MSC, 2004 ▶); program(s) used to solve structure: *SIR2004* (Burla *et al.*, 2005 ▶); program(s) used to refine structure: *SHELXL97* (Sheldrick, 2008 ▶); molecular graphics: *ORTEP-3 for Windows* (Farrugia, 1997 ▶); software used to prepare material for publication: *SHELXL97*.

## Supplementary Material

Crystal structure: contains datablocks I, global. DOI: 10.1107/S1600536809028591/dn2461sup1.cif

Structure factors: contains datablocks I. DOI: 10.1107/S1600536809028591/dn2461Isup2.hkl

Additional supplementary materials:  crystallographic information; 3D view; checkCIF report

## Figures and Tables

**Fig. 1.**
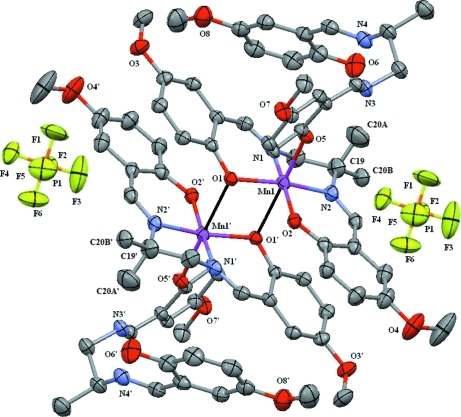
A view of the title compound, showing the atom-numbering scheme. Displacement ellipsoids are drawn at the 30% probability level and The hydrogen atoms are omitted for clarity. [Symmetry code: (') 1-x, 1-y, 1-z]

## supplementary crystallographic information

### Comment

Schiff base ligands with nitrogen and oxygen donor atoms seem to stabilize the various oxidation states of manganese better than any other ligand systems, as it is evident from the sheer number of publications in this area (Vites & Lynam, 1998). The penta-coordinated [Mn(salen)Cl] (H2salen = N,N'-bis(salicylidene)-1,2-diaminoethane) was one of the earliest crystallographically characterized manganese(III) Schiff base complexes (Pecoraro & Butler, 1986).

Crystallographic studies on the active sites of a relatively rare class of manganese catalases found in bacteria-like Thermus thermophilus and Lactobacillus plantarum point to a dinuclear manganese core with an Mn···Mn separation of 3.13 Å (reduced state) and 3.03 Å (oxidized state) respectively (Antonyuk *et al.*, 2000; Barynin *et al.*, 2001). The Mn···Mn distances derived from the EPR and EXAFS data provide complementary structural parameters with the Mn···Mn distances being 3.4 Å and 3.54 Å, respectively (Meier *et al.*, 1996; Stemmler *et al.*, 1997).Here we report the crystal structure of a dimeric manganese complex with a Mn···Mn distance of 3.451 (2) Å, I (Figure 1).

Manganese complexes in various oxidation states and in various combinations of nitrogen and oxygen donor environment have been used as models for oxygen-evolving complex of photosystem II (Glatzel *et al.*, 2004), catalysis (Dixit & Srinivasan, 1988), single-molecule magnet (Lu *et al.*, 2006) and as active sites of manganese-containing metal enzymes (Stallings *et al.*, 1985).

Recently, we reported the crystal structure Mn^III^, Cu^II^ and Ni^II^ with Schiff base ligands (Habibi *et al.*, 2007*a*,b,c) and herein the crystal structure of the Mn^III^ complex with *N*,*N*'-bis(5-methoxysalicylidene)-1,2-diimino-propane is reported. In (I), two manganese(III) ions, which are in slightly distorted octahedral environments, are linked by phenoxy bridges using the phenolic oxygen atoms of each ligand. The formation of the phenoxy bridges and the nearly planar nature of the tetradentate Schiff base ligand lead the hydroxy group of a free ligand to adopt a relatively rare unidentate bonding mode. There are two stereogenic centers C39 and C19. However, the methyl group and H atom attached to C19 are disordered over two positions with occupancy factors in the ratio 0.58/0.42. So, the crystal is a mixture of two diasteroisomers, *RS/SR* and *RR/SS*.

The Mn—O distances [Mn1—O1 = 1.887 (2) Å, Mn1—O2 = 1.855 (2) Å] and Mn—N distances [Mn1—N1 = 1.982 (3) Å, Mn1—N2 = 1.977 (3) Å] are in the same ranges as those observed in other related Mn^III ^complexes of N_2_O_2_ Schiff base ligands (Eltayeb *et al.*, 2008*a*,b; Mitra *et al.*, 2006). An axial elongation, of the Mn–O Hydroxy bond [Mn1–O5 = 2.129 (2)Å], nearly orthogonal to the plane of the Schiff base, is indicative of the Jahn-Teller distortion anticipated of a high-spin manganese(III) ion in octahedral surroundings. This also causes a considerable weakening of the Mn–O bond along the phenoxy bridge [Mn1-O1' = 2.634 (2)Å], leading to an asymmetric Mn1–O–Mn1' bridge. Other bond lengths and angles observed in the structure are also normal (Allen *et al.*, 1987).

### Experimental

To a stirring solution of Mn(CH_3_COO)_2_.2H_2_O (0.0662 g, 0.5 mmol) in methanol (25 ml) was added an 1 mmol of (2-hydroxy5-methoxy banzenaldehyde and 1,2 diamino propane (0.342 g). The pinksolution turned dark brown immediately upon the formation of Mn^II^complex. 0.5 mmol of NH_4_PF_6 _was then added to the resulting dark brown solution and stirred for 5 minutes. A dark brown microcrystalline solid was produced by slow evaporation ofmethanol at room temperature. The product was then recrystallized from methanol-propanol (2:1 *v*/*v*) and dark brown crystals suitable for X-raycrystallography were obtained (m.p = 256 °c).

### Refinement

All H atoms were fixed geometrically and treated as riding on their parent C atoms with C—H = 0.93Å (aromatic), 0.96 Å (methyl), 0.97 Å (methylene) and 0.98\ %A (methine) and with U_iso_(H) = 1.2U_eq_(C) or U_iso_(H) = 1.5U_eq_(methyl).

The disordered methyl C20A and C20B were treated using the tools (PART, EXYZ, EADP and DFIX) available in SHELXL-97 (Sheldrick, 2008)

### Crystal data

**Table:**

[Mn_2_(C_19_H_20_N_2_O_4_)_2_(C_19_H_22_N_2_O_4_)_2_](PF_6_)_2_	*F*(000) = 1816
*M**_r_* = 1761.30	*D*_x_ = 1.484 Mg m^−^^3^
Monoclinic, *P*2_1_/*n*	Mo *K*α radiation, λ = 0.71075 Å
Hall symbol: -P 2yn	Cell parameters from 36538 reflections
*a* = 13.2754 (5) Å	θ = 3.1–27.6°
*b* = 22.658 (1) Å	µ = 0.46 mm^−^^1^
*c* = 14.0774 (5) Å	*T* = 200 K
β = 111.476 (1)°	Platelet, dark purple
*V* = 3940.4 (3) Å^3^	0.26 × 0.12 × 0.08 mm
*Z* = 2	

### Data collection

**Table:**

Rigaku R-AXIS RAPID diffractometer	8971 independent reflections
Radiation source: fine-focus sealed tube	5923 reflections with *I* > 2σ(*I*)
graphite	*R*_int_ = 0.054
Detector resolution: 10.00 pixels mm^-1^	θ_max_ = 27.5°, θ_min_ = 3.1°
ω scans	*h* = −17→17
Absorption correction: numerical (*ABSCOR*; Higashi, 1995)	*k* = −29→29
*T*_min_ = 0.890, *T*_max_ = 0.964	*l* = −18→18
60946 measured reflections	

### Refinement

**Table:**

Refinement on *F*^2^	Primary atom site location: structure-invariant direct methods
Least-squares matrix: full	Secondary atom site location: difference Fourier map
*R*[*F*^2^ > 2σ(*F*^2^)] = 0.070	Hydrogen site location: inferred from neighbouring sites
*wR*(*F*^2^) = 0.219	H-atom parameters constrained
*S* = 1.08	*w* = 1/[σ^2^(*F*_o_^2^) + (0.1176*P*)^2^ + 2.1813*P*] where *P* = (*F*_o_^2^ + 2*F*_c_^2^)/3
8971 reflections	(Δ/σ)_max_ = 0.002
539 parameters	Δρ_max_ = 0.73 e Å^−^^3^
2 restraints	Δρ_min_ = −0.62 e Å^−^^3^

### Special details

**Table:**

Geometry. All esds (except the esd in the dihedral angle between two l.s. planes) are estimated using the full covariance matrix. The cell esds are taken into account individually in the estimation of esds in distances, angles and torsion angles; correlations between esds in cell parameters are only used when they are defined by crystal symmetry. An approximate (isotropic) treatment of cell esds is used for estimating esds involving l.s. planes.
Refinement. Refinement of *F*^2^ against ALL reflections. The weighted *R*-factor *wR* and goodness of fit *S* are based on *F*^2^, conventional *R*-factors *R* are based on *F*, with *F* set to zero for negative *F*^2^. The threshold expression of *F*^2^ > σ(*F*^2^) is used only for calculating *R*-factors(gt) *etc*. and is not relevant to the choice of reflections for refinement. *R*-factors based on *F*^2^ are statistically about twice as large as those based on *F*, and *R*- factors based on ALL data will be even larger.

### Fractional atomic coordinates and isotropic or equivalent isotropic displacement parameters (Å^2^)

**Table:**

	*x*	*y*	*z*	*U*_iso_*/*U*_eq_	Occ. (<1)
Mn1	0.50832 (4)	0.54235 (2)	0.40128 (4)	0.04105 (18)	
P1	0.85574 (13)	0.21346 (6)	0.65195 (12)	0.0802 (4)	
O1	0.57882 (18)	0.47151 (9)	0.45950 (17)	0.0408 (5)	
O2	0.38872 (19)	0.50383 (10)	0.30998 (18)	0.0464 (5)	
O3	1.0178 (2)	0.43197 (14)	0.6173 (2)	0.0669 (8)	
O4	−0.0250 (3)	0.5749 (2)	0.0818 (3)	0.1104 (15)	
O5	0.5854 (2)	0.55631 (10)	0.29423 (18)	0.0468 (6)	
O6	0.7419 (3)	0.77319 (16)	0.4936 (3)	0.0909 (11)	
O7	0.6231 (2)	0.39559 (12)	0.0143 (2)	0.0601 (7)	
O8	1.0484 (3)	0.61541 (16)	0.7263 (3)	0.0824 (9)	
N1	0.6205 (2)	0.58638 (12)	0.5116 (2)	0.0468 (7)	
N2	0.4319 (3)	0.61889 (13)	0.3818 (2)	0.0510 (7)	
N3	0.6265 (3)	0.64129 (14)	0.1882 (2)	0.0545 (8)	
N4	0.7710 (3)	0.72855 (14)	0.3342 (3)	0.0590 (8)	
C1	0.6873 (3)	0.46627 (14)	0.4941 (2)	0.0397 (7)	
C2	0.7313 (3)	0.41140 (15)	0.4848 (3)	0.0456 (8)	
H2	0.6857	0.3811	0.4495	0.055*	
C3	0.8407 (3)	0.40178 (17)	0.5270 (3)	0.0525 (9)	
H3	0.8686	0.3649	0.5214	0.063*	
C4	0.9108 (3)	0.44740 (17)	0.5788 (3)	0.0516 (9)	
C5	0.8698 (3)	0.50179 (16)	0.5868 (3)	0.0466 (8)	
H5	0.9163	0.5320	0.6211	0.056*	
C6	0.7580 (3)	0.51222 (14)	0.5437 (3)	0.0429 (7)	
C7	0.7173 (3)	0.56869 (16)	0.5596 (3)	0.0478 (8)	
H7	0.7643	0.5939	0.6077	0.057*	
C8	1.0930 (4)	0.4760 (2)	0.6710 (4)	0.0789 (14)	
H8A	1.0838	0.5103	0.6286	0.118*	
H8B	1.1653	0.4611	0.6887	0.118*	
H8C	1.0809	0.4864	0.7321	0.118*	
C9	0.5790 (4)	0.64073 (18)	0.5408 (4)	0.0650 (11)	
H9A	0.6379	0.6680	0.5734	0.078*	
H9B	0.5433	0.6319	0.5881	0.078*	
C11	0.2910 (3)	0.52508 (17)	0.2556 (3)	0.0490 (8)	
C12	0.2160 (3)	0.48661 (19)	0.1878 (3)	0.0560 (9)	
H12	0.2363	0.4480	0.1812	0.067*	
C13	0.1140 (4)	0.5050 (2)	0.1317 (3)	0.0707 (12)	
H13	0.0656	0.4790	0.0866	0.085*	
C14	0.0808 (4)	0.5631 (3)	0.1411 (3)	0.0749 (13)	
C15	0.1523 (4)	0.6012 (2)	0.2055 (3)	0.0646 (11)	
H15	0.1306	0.6395	0.2116	0.078*	
C16	0.2595 (3)	0.58344 (17)	0.2636 (3)	0.0502 (8)	
C17	0.3304 (3)	0.62637 (16)	0.3275 (3)	0.0524 (9)	
H17	0.3011	0.6633	0.3304	0.063*	
C18	−0.0593 (6)	0.6329 (4)	0.0890 (6)	0.163 (4)	
H18A	−0.0511	0.6411	0.1584	0.244*	
H18B	−0.1339	0.6372	0.0456	0.244*	
H18C	−0.0161	0.6602	0.0679	0.244*	
C19A	0.4990 (4)	0.66759 (18)	0.4431 (4)	0.0745 (13)	0.58
H19A	0.5435	0.6804	0.4047	0.089*	0.58
C20A	0.4552 (7)	0.7222 (3)	0.4682 (7)	0.076 (2)	0.58
H20A	0.4161	0.7433	0.4065	0.114*	0.58
H20B	0.5136	0.7463	0.5113	0.114*	0.58
H20C	0.4074	0.7129	0.5033	0.114*	0.58
C19B	0.4990 (4)	0.66759 (18)	0.4431 (4)	0.0745 (13)	0.42
H19B	0.4517	0.6948	0.4615	0.089*	0.42
C20B	0.5484 (11)	0.6983 (5)	0.3871 (11)	0.090 (4)	0.42
H20D	0.6031	0.6740	0.3774	0.135*	0.42
H20E	0.5811	0.7335	0.4232	0.135*	0.42
H20F	0.4953	0.7087	0.3219	0.135*	0.42
C21	0.5934 (3)	0.51903 (15)	0.2261 (3)	0.0428 (7)	
C22	0.5703 (3)	0.45811 (15)	0.2278 (3)	0.0450 (8)	
H22	0.5471	0.4437	0.2781	0.054*	
C23	0.5816 (3)	0.42025 (16)	0.1573 (3)	0.0475 (8)	
H23	0.5659	0.3805	0.1609	0.057*	
C24	0.6163 (3)	0.43960 (16)	0.0787 (3)	0.0472 (8)	
C25	0.6349 (3)	0.49776 (16)	0.0707 (3)	0.0465 (8)	
H25	0.6553	0.5112	0.0179	0.056*	
C26	0.6232 (3)	0.53851 (15)	0.1434 (3)	0.0435 (7)	
C27	0.6347 (3)	0.59874 (17)	0.1272 (3)	0.0506 (8)	
H27	0.6490	0.6095	0.0696	0.061*	
C28	0.6579 (4)	0.4119 (2)	−0.0662 (3)	0.0636 (11)	
H28A	0.6087	0.4404	−0.1092	0.095*	
H28B	0.6594	0.3776	−0.1057	0.095*	
H28C	0.7291	0.4287	−0.0381	0.095*	
C29	0.6360 (4)	0.70408 (18)	0.1668 (3)	0.0617 (10)	
H29A	0.5851	0.7268	0.1868	0.074*	
H29B	0.6181	0.7096	0.0941	0.074*	
C34	0.9724 (3)	0.65620 (19)	0.6738 (3)	0.0612 (10)	
C33	0.9172 (4)	0.6930 (2)	0.7160 (4)	0.0745 (13)	
H33	0.9314	0.6913	0.7858	0.089*	
C32	0.8416 (4)	0.7319 (2)	0.6559 (4)	0.0745 (13)	
H32	0.8061	0.7568	0.6859	0.089*	
C31	0.8165 (4)	0.73497 (19)	0.5504 (4)	0.0643 (11)	
C36	0.8711 (3)	0.69699 (17)	0.5065 (3)	0.0550 (9)	
C35	0.9476 (3)	0.65829 (18)	0.5691 (3)	0.0575 (9)	
H35	0.9834	0.6329	0.5401	0.069*	
C37	0.8454 (3)	0.69691 (17)	0.3951 (3)	0.0552 (9)	
H37	0.8853	0.6727	0.3687	0.066*	
C38	1.0812 (5)	0.6140 (3)	0.8338 (4)	0.106 (2)	
H38A	1.1194	0.6498	0.8620	0.159*	
H38B	1.1280	0.5808	0.8601	0.159*	
H38C	1.0186	0.6107	0.8522	0.159*	
C39	0.7505 (4)	0.72634 (18)	0.2248 (3)	0.0604 (10)	
H39	0.8024	0.6993	0.2130	0.073*	
C40	0.7625 (5)	0.7869 (2)	0.1846 (4)	0.0841 (15)	
H40A	0.7120	0.8136	0.1962	0.126*	
H40B	0.7480	0.7844	0.1128	0.126*	
H40C	0.8349	0.8010	0.2194	0.126*	
F1	0.9469 (4)	0.1776 (2)	0.7337 (3)	0.1372 (16)	
F2	0.8327 (4)	0.1668 (2)	0.5674 (4)	0.1596 (19)	
F3	0.7669 (4)	0.1813 (3)	0.6821 (4)	0.178 (2)	
F4	0.8558 (4)	0.2556 (2)	0.7402 (4)	0.1608 (19)	
F5	0.7713 (7)	0.2535 (3)	0.5751 (5)	0.258 (4)	
F6	0.9398 (5)	0.2407 (5)	0.6197 (6)	0.302 (6)	

### Atomic displacement parameters (Å^2^)

**Table:**

	*U*^11^	*U*^22^	*U*^33^	*U*^12^	*U*^13^	*U*^23^
Mn1	0.0493 (3)	0.0322 (3)	0.0468 (3)	0.0018 (2)	0.0237 (2)	−0.0002 (2)
P1	0.0924 (10)	0.0646 (8)	0.0917 (9)	0.0094 (7)	0.0432 (8)	−0.0076 (7)
O1	0.0445 (12)	0.0344 (11)	0.0472 (13)	−0.0001 (9)	0.0212 (10)	−0.0005 (9)
O2	0.0505 (13)	0.0401 (12)	0.0496 (13)	0.0060 (10)	0.0195 (11)	0.0004 (10)
O3	0.0451 (15)	0.0668 (18)	0.086 (2)	0.0062 (13)	0.0207 (14)	−0.0128 (15)
O4	0.077 (2)	0.131 (4)	0.088 (3)	0.042 (2)	−0.010 (2)	−0.019 (2)
O5	0.0595 (15)	0.0395 (12)	0.0494 (13)	−0.0020 (11)	0.0296 (12)	−0.0001 (10)
O6	0.109 (3)	0.082 (2)	0.093 (2)	0.038 (2)	0.051 (2)	−0.0036 (19)
O7	0.088 (2)	0.0548 (15)	0.0507 (14)	0.0082 (14)	0.0407 (15)	0.0001 (12)
O8	0.083 (2)	0.084 (2)	0.068 (2)	0.0030 (18)	0.0132 (17)	0.0008 (17)
N1	0.0568 (18)	0.0338 (14)	0.0583 (17)	0.0003 (12)	0.0310 (15)	−0.0043 (12)
N2	0.0587 (19)	0.0377 (15)	0.0635 (19)	0.0077 (13)	0.0304 (16)	0.0021 (13)
N3	0.065 (2)	0.0424 (16)	0.0576 (18)	−0.0042 (14)	0.0240 (16)	0.0071 (14)
N4	0.074 (2)	0.0440 (17)	0.064 (2)	−0.0029 (16)	0.0315 (18)	0.0003 (15)
C1	0.0484 (18)	0.0372 (16)	0.0415 (16)	0.0018 (13)	0.0258 (15)	0.0017 (13)
C2	0.0504 (19)	0.0400 (17)	0.053 (2)	−0.0009 (14)	0.0270 (16)	−0.0060 (15)
C3	0.054 (2)	0.0464 (19)	0.067 (2)	0.0077 (16)	0.0339 (19)	−0.0029 (17)
C4	0.047 (2)	0.056 (2)	0.057 (2)	0.0067 (16)	0.0261 (17)	−0.0010 (17)
C5	0.0486 (19)	0.0462 (19)	0.0499 (19)	−0.0033 (15)	0.0238 (16)	−0.0045 (15)
C6	0.0528 (19)	0.0375 (17)	0.0455 (18)	−0.0018 (14)	0.0264 (16)	−0.0021 (14)
C7	0.053 (2)	0.0433 (18)	0.055 (2)	−0.0058 (15)	0.0287 (18)	−0.0083 (15)
C8	0.047 (2)	0.083 (3)	0.093 (4)	0.002 (2)	0.011 (2)	−0.012 (3)
C9	0.067 (3)	0.044 (2)	0.083 (3)	−0.0001 (18)	0.027 (2)	−0.022 (2)
C11	0.059 (2)	0.055 (2)	0.0385 (17)	0.0032 (17)	0.0247 (17)	0.0058 (15)
C12	0.059 (2)	0.060 (2)	0.047 (2)	0.0063 (19)	0.0172 (18)	0.0039 (18)
C13	0.063 (3)	0.088 (3)	0.052 (2)	0.006 (2)	0.010 (2)	−0.003 (2)
C14	0.067 (3)	0.095 (4)	0.056 (2)	0.027 (3)	0.015 (2)	0.003 (2)
C15	0.073 (3)	0.070 (3)	0.052 (2)	0.022 (2)	0.025 (2)	0.003 (2)
C16	0.057 (2)	0.057 (2)	0.0433 (18)	0.0152 (17)	0.0255 (17)	0.0108 (16)
C17	0.065 (2)	0.0437 (19)	0.058 (2)	0.0135 (17)	0.034 (2)	0.0094 (16)
C18	0.107 (5)	0.191 (9)	0.136 (6)	0.091 (6)	−0.021 (5)	−0.040 (6)
C19A	0.081 (3)	0.037 (2)	0.106 (4)	0.005 (2)	0.035 (3)	−0.009 (2)
C20A	0.081 (5)	0.053 (4)	0.098 (6)	0.006 (4)	0.037 (5)	0.002 (4)
C19B	0.081 (3)	0.037 (2)	0.106 (4)	0.005 (2)	0.035 (3)	−0.009 (2)
C20B	0.094 (9)	0.061 (7)	0.125 (11)	−0.019 (6)	0.052 (8)	0.001 (7)
C21	0.0442 (18)	0.0442 (17)	0.0430 (17)	0.0023 (14)	0.0195 (15)	0.0028 (14)
C22	0.057 (2)	0.0415 (17)	0.0453 (18)	0.0022 (15)	0.0291 (16)	0.0055 (14)
C23	0.057 (2)	0.0398 (17)	0.051 (2)	0.0016 (15)	0.0254 (17)	0.0035 (15)
C24	0.055 (2)	0.0486 (19)	0.0417 (18)	0.0067 (16)	0.0215 (16)	0.0014 (15)
C25	0.0498 (19)	0.055 (2)	0.0407 (17)	0.0016 (16)	0.0242 (15)	0.0088 (15)
C26	0.0457 (18)	0.0458 (18)	0.0423 (17)	−0.0020 (14)	0.0200 (15)	0.0069 (14)
C27	0.056 (2)	0.053 (2)	0.0464 (19)	−0.0053 (17)	0.0232 (17)	0.0060 (16)
C28	0.079 (3)	0.077 (3)	0.045 (2)	0.015 (2)	0.035 (2)	0.0054 (19)
C29	0.073 (3)	0.051 (2)	0.064 (2)	−0.0044 (19)	0.028 (2)	0.0111 (19)
C34	0.061 (2)	0.057 (2)	0.068 (3)	−0.0103 (19)	0.026 (2)	−0.005 (2)
C33	0.086 (3)	0.082 (3)	0.064 (3)	−0.017 (3)	0.037 (3)	−0.013 (2)
C32	0.093 (3)	0.070 (3)	0.076 (3)	0.003 (3)	0.048 (3)	−0.017 (2)
C31	0.073 (3)	0.058 (2)	0.072 (3)	0.004 (2)	0.039 (2)	−0.008 (2)
C36	0.063 (2)	0.049 (2)	0.064 (2)	−0.0063 (17)	0.037 (2)	−0.0069 (17)
C35	0.059 (2)	0.055 (2)	0.065 (2)	−0.0040 (18)	0.030 (2)	−0.0049 (19)
C37	0.064 (2)	0.046 (2)	0.068 (2)	−0.0074 (18)	0.039 (2)	−0.0068 (18)
C38	0.111 (5)	0.102 (5)	0.078 (4)	−0.008 (4)	0.004 (3)	0.000 (3)
C39	0.072 (3)	0.049 (2)	0.067 (2)	−0.0086 (19)	0.034 (2)	0.0060 (18)
C40	0.095 (4)	0.065 (3)	0.091 (4)	−0.017 (3)	0.033 (3)	0.008 (3)
F1	0.133 (3)	0.162 (4)	0.120 (3)	0.068 (3)	0.049 (3)	0.009 (3)
F2	0.187 (4)	0.158 (4)	0.158 (4)	−0.032 (3)	0.092 (4)	−0.082 (3)
F3	0.160 (4)	0.246 (6)	0.175 (4)	−0.074 (4)	0.117 (4)	−0.061 (4)
F4	0.178 (4)	0.121 (3)	0.187 (4)	0.030 (3)	0.072 (4)	−0.050 (3)
F5	0.340 (10)	0.207 (7)	0.176 (5)	0.159 (7)	0.034 (6)	0.034 (5)
F6	0.155 (5)	0.434 (13)	0.298 (9)	−0.117 (7)	0.060 (5)	0.169 (9)

### Geometric parameters (Å, °)

**Table:**

Mn1—O2	1.855 (2)	C13—H13	0.9300
Mn1—O1	1.887 (2)	C14—C15	1.355 (7)
Mn1—N2	1.977 (3)	C15—C16	1.416 (6)
Mn1—N1	1.983 (3)	C15—H15	0.9300
Mn1—O5	2.133 (2)	C16—C17	1.421 (6)
Mn1—O1^i^	2.634 (2)	C17—H17	0.9300
P1—F6	1.484 (5)	C18—H18A	0.9600
P1—F2	1.536 (4)	C18—H18B	0.9600
P1—F5	1.537 (6)	C18—H18C	0.9600
P1—F1	1.559 (4)	C19A—C20A	1.464 (8)
P1—F4	1.566 (4)	C19A—H19A	0.9800
P1—F3	1.572 (5)	C20A—H20A	0.9600
O1—C1	1.346 (4)	C20A—H20B	0.9600
O2—C11	1.332 (4)	C20A—H20C	0.9600
O3—C4	1.368 (4)	C20B—H20D	0.9600
O3—C8	1.418 (6)	C20B—H20E	0.9600
O4—C14	1.372 (6)	C20B—H20F	0.9600
O4—C18	1.407 (8)	C21—C22	1.416 (5)
O5—C21	1.311 (4)	C21—C26	1.430 (5)
O6—C31	1.338 (6)	C22—C23	1.360 (5)
O7—C24	1.373 (4)	C22—H22	0.9300
O7—C28	1.421 (4)	C23—C24	1.415 (5)
O8—C34	1.369 (5)	C23—H23	0.9300
O8—C38	1.413 (7)	C24—C25	1.353 (5)
N1—C7	1.278 (5)	C25—C26	1.429 (5)
N1—C9	1.468 (5)	C25—H25	0.9300
N2—C17	1.294 (5)	C26—C27	1.401 (5)
N2—C19A	1.481 (6)	C27—H27	0.9300
N3—C27	1.322 (5)	C28—H28A	0.9600
N3—C29	1.469 (5)	C28—H28B	0.9600
N4—C37	1.266 (5)	C28—H28C	0.9600
N4—C39	1.463 (5)	C29—C39	1.524 (6)
C1—C2	1.400 (4)	C29—H29A	0.9700
C1—C6	1.405 (5)	C29—H29B	0.9700
C2—C3	1.371 (5)	C34—C33	1.379 (6)
C2—H2	0.9300	C34—C35	1.388 (6)
C3—C4	1.403 (5)	C33—C32	1.371 (7)
C3—H3	0.9300	C33—H33	0.9300
C4—C5	1.369 (5)	C32—C31	1.400 (6)
C5—C6	1.402 (5)	C32—H32	0.9300
C5—H5	0.9300	C31—C36	1.405 (5)
C6—C7	1.438 (5)	C36—C35	1.386 (6)
C7—H7	0.9300	C36—C37	1.479 (5)
C8—H8A	0.9600	C35—H35	0.9300
C8—H8B	0.9600	C37—H37	0.9300
C8—H8C	0.9600	C38—H38A	0.9600
C9—C19A	1.523 (7)	C38—H38B	0.9600
C9—H9A	0.9700	C38—H38C	0.9600
C9—H9B	0.9700	C39—C40	1.514 (6)
C11—C12	1.402 (6)	C39—H39	0.9800
C11—C16	1.404 (5)	C40—H40A	0.9600
C12—C13	1.360 (6)	C40—H40B	0.9600
C12—H12	0.9300	C40—H40C	0.9600
C13—C14	1.409 (7)		
			
O2—Mn1—O1	93.63 (10)	C11—C16—C15	119.1 (4)
O2—Mn1—N2	92.93 (12)	C11—C16—C17	123.2 (3)
O1—Mn1—N2	162.53 (11)	C15—C16—C17	117.7 (4)
O2—Mn1—N1	170.57 (11)	N2—C17—C16	126.1 (3)
O1—Mn1—N1	88.68 (11)	N2—C17—H17	117.0
N2—Mn1—N1	82.38 (13)	C16—C17—H17	117.0
O2—Mn1—O5	95.20 (10)	O4—C18—H18A	109.5
O1—Mn1—O5	98.23 (9)	O4—C18—H18B	109.5
N2—Mn1—O5	97.28 (11)	H18A—C18—H18B	109.5
N1—Mn1—O5	93.51 (11)	O4—C18—H18C	109.5
O1—Mn1—O1^i^	81.87 (9)	H18A—C18—H18C	109.5
O1^i^—Mn1—O2	87.81 (9)	H18B—C18—H18C	109.5
O1^i^—Mn1—O5	176.98 (9)	C20A—C19A—N2	124.3 (5)
O1^i^—Mn1—N1	83.44 (10)	C20A—C19A—C9	109.1 (5)
O1^i^—Mn1—N2	82.24 (11)	N2—C19A—C9	107.5 (3)
F6—P1—F2	90.5 (5)	C20A—C19A—H19A	104.8
F6—P1—F5	88.6 (5)	N2—C19A—H19A	104.8
F2—P1—F5	88.5 (4)	C9—C19A—H19A	104.8
F6—P1—F1	88.6 (4)	H20D—C20B—H20E	109.5
F2—P1—F1	95.4 (3)	H20D—C20B—H20F	109.5
F5—P1—F1	175.2 (4)	H20E—C20B—H20F	109.5
F6—P1—F4	101.9 (5)	O5—C21—C22	122.7 (3)
F2—P1—F4	167.3 (3)	O5—C21—C26	121.1 (3)
F5—P1—F4	89.2 (3)	C22—C21—C26	116.1 (3)
F1—P1—F4	87.6 (3)	C23—C22—C21	121.4 (3)
F6—P1—F3	176.6 (5)	C23—C22—H22	119.3
F2—P1—F3	86.2 (3)	C21—C22—H22	119.3
F5—P1—F3	92.2 (5)	C22—C23—C24	121.9 (3)
F1—P1—F3	90.8 (3)	C22—C23—H23	119.0
F4—P1—F3	81.4 (3)	C24—C23—H23	119.0
C1—O1—Mn1	121.79 (19)	C25—C24—O7	126.5 (3)
C11—O2—Mn1	129.4 (2)	C25—C24—C23	119.3 (3)
C4—O3—C8	117.3 (3)	O7—C24—C23	114.2 (3)
C14—O4—C18	115.1 (5)	C24—C25—C26	119.9 (3)
C21—O5—Mn1	127.5 (2)	C24—C25—H25	120.1
C24—O7—C28	117.2 (3)	C26—C25—H25	120.1
C34—O8—C38	118.0 (4)	C27—C26—C25	117.8 (3)
C7—N1—C9	121.5 (3)	C27—C26—C21	120.8 (3)
C7—N1—Mn1	125.3 (2)	C25—C26—C21	121.3 (3)
C9—N1—Mn1	113.0 (2)	N3—C27—C26	124.4 (3)
C17—N2—C19A	121.4 (3)	N3—C27—H27	117.8
C17—N2—Mn1	124.6 (3)	C26—C27—H27	117.8
C19A—N2—Mn1	113.8 (3)	O7—C28—H28A	109.5
C27—N3—C29	122.7 (3)	O7—C28—H28B	109.5
C37—N4—C39	119.4 (4)	H28A—C28—H28B	109.5
O1—C1—C2	118.2 (3)	O7—C28—H28C	109.5
O1—C1—C6	123.1 (3)	H28A—C28—H28C	109.5
C2—C1—C6	118.7 (3)	H28B—C28—H28C	109.5
C3—C2—C1	120.8 (3)	N3—C29—C39	110.8 (3)
C3—C2—H2	119.6	N3—C29—H29A	109.5
C1—C2—H2	119.6	C39—C29—H29A	109.5
C2—C3—C4	120.3 (3)	N3—C29—H29B	109.5
C2—C3—H3	119.9	C39—C29—H29B	109.5
C4—C3—H3	119.9	H29A—C29—H29B	108.1
O3—C4—C5	125.7 (4)	O8—C34—C33	125.3 (4)
O3—C4—C3	114.4 (3)	O8—C34—C35	115.9 (4)
C5—C4—C3	119.9 (3)	C33—C34—C35	118.7 (4)
C4—C5—C6	120.4 (3)	C32—C33—C34	120.5 (4)
C4—C5—H5	119.8	C32—C33—H33	119.8
C6—C5—H5	119.8	C34—C33—H33	119.8
C5—C6—C1	119.9 (3)	C33—C32—C31	121.5 (4)
C5—C6—C7	118.8 (3)	C33—C32—H32	119.2
C1—C6—C7	121.1 (3)	C31—C32—H32	119.2
N1—C7—C6	124.0 (3)	O6—C31—C32	120.2 (4)
N1—C7—H7	118.0	O6—C31—C36	121.5 (4)
C6—C7—H7	118.0	C32—C31—C36	118.4 (4)
O3—C8—H8A	109.5	C35—C36—C31	118.9 (4)
O3—C8—H8B	109.5	C35—C36—C37	120.3 (3)
H8A—C8—H8B	109.5	C31—C36—C37	120.7 (4)
O3—C8—H8C	109.5	C36—C35—C34	122.0 (4)
H8A—C8—H8C	109.5	C36—C35—H35	119.0
H8B—C8—H8C	109.5	C34—C35—H35	119.0
N1—C9—C19A	106.9 (3)	N4—C37—C36	122.5 (4)
N1—C9—H9A	110.3	N4—C37—H37	118.7
C19A—C9—H9A	110.3	C36—C37—H37	118.7
N1—C9—H9B	110.3	O8—C38—H38A	109.5
C19A—C9—H9B	110.3	O8—C38—H38B	109.5
H9A—C9—H9B	108.6	H38A—C38—H38B	109.5
O2—C11—C12	117.8 (3)	O8—C38—H38C	109.5
O2—C11—C16	123.4 (3)	H38A—C38—H38C	109.5
C12—C11—C16	118.8 (4)	H38B—C38—H38C	109.5
C13—C12—C11	120.8 (4)	N4—C39—C40	110.9 (4)
C13—C12—H12	119.6	N4—C39—C29	109.8 (3)
C11—C12—H12	119.6	C40—C39—C29	108.7 (4)
C12—C13—C14	120.7 (4)	N4—C39—H39	109.1
C12—C13—H13	119.6	C40—C39—H39	109.1
C14—C13—H13	119.6	C29—C39—H39	109.1
C15—C14—O4	126.3 (5)	C39—C40—H40A	109.5
C15—C14—C13	119.4 (4)	C39—C40—H40B	109.5
O4—C14—C13	114.3 (5)	H40A—C40—H40B	109.5
C14—C15—C16	121.1 (4)	C39—C40—H40C	109.5
C14—C15—H15	119.5	H40A—C40—H40C	109.5
C16—C15—H15	119.5	H40B—C40—H40C	109.5

Symmetry codes: (i) −*x*+1, −*y*+1, −*z*+1.
